# P-1923. Wide Geographical Distribution and Long Delayed Diagnoses of Chromoblastomycosis in the United States: A Literature Review

**DOI:** 10.1093/ofid/ofaf695.2092

**Published:** 2026-01-11

**Authors:** Alexa G Ries, Nicole Boswell, Jamie Wong, Karolyn Wanat, Eva Rawlings Parker, Dallas J Smith

**Affiliations:** Medical College of Wisconsin, Milwaukee, WI; Medical College of Wisconsin, Milwaukee, WI; Long Island University, New York, New York; Medical College of Wisconsin, Milwaukee, WI; Vanderbilt University Medical Center, Nashville, Tennessee; Mycotic Diseases Branch, Centers for Disease Control and Prevention, Atlanta, Georgia

## Abstract

**Background:**

Chromoblastomycosis (CBM) is a fungal neglected tropical disease acquired via traumatic inoculation and characterized by chronic granulomatous inflammation of the skin and subcutaneous tissue. CBM is associated with poverty and most often occurs in tropical and subtropical regions; sporadic cases occur in the United States. Prior systematic reviews of CBM lacked information on inoculation, clinical presentation, or treatment for U.S. cases.^1^ We conducted a systematic review to better characterize CBM in the United States and inform clinical and public health interventions.

**Table 1:**
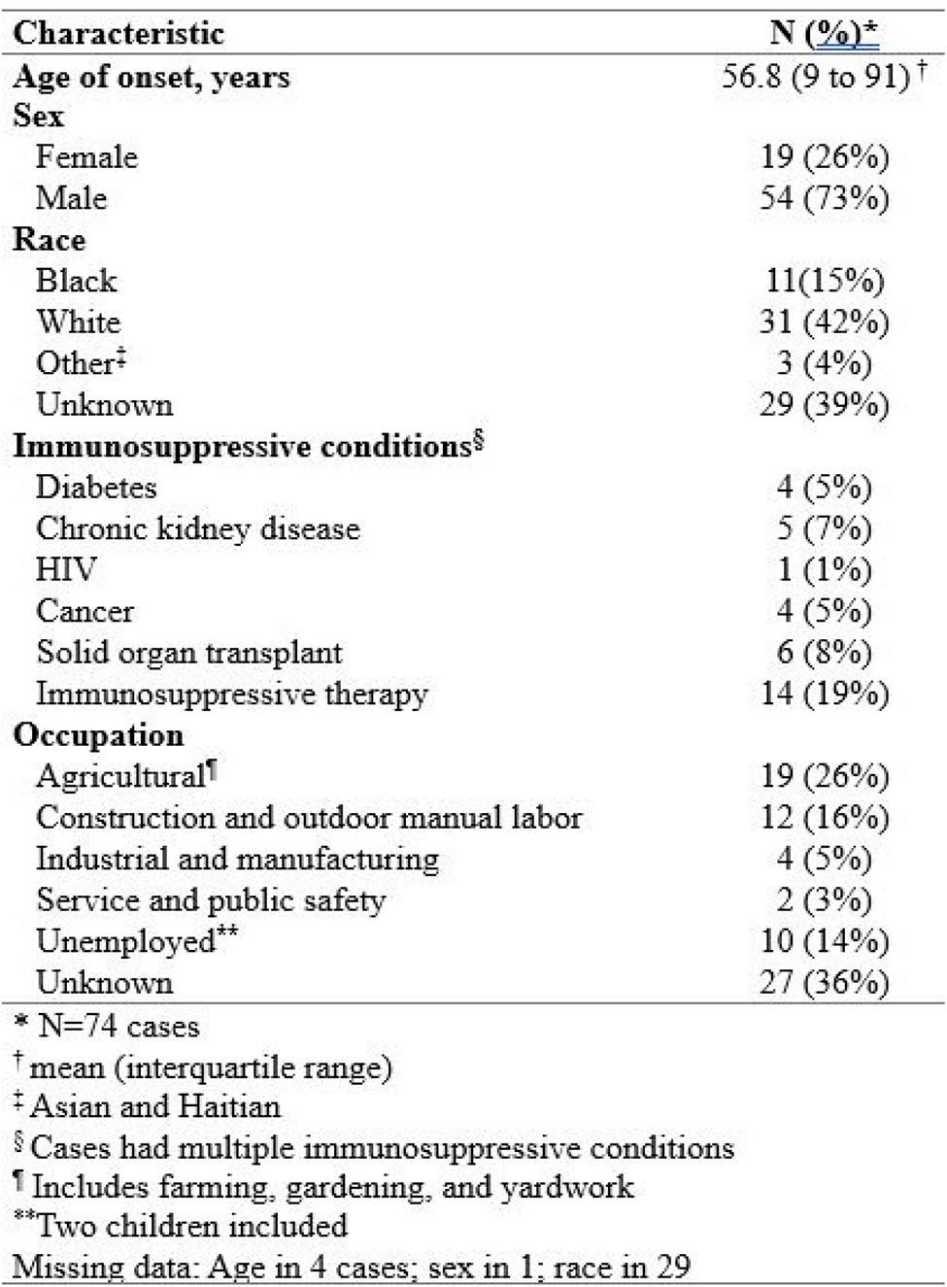
Clinical demographics of chromoblastomycosis cases in the United States and Puerto Rico

**Table 2:**
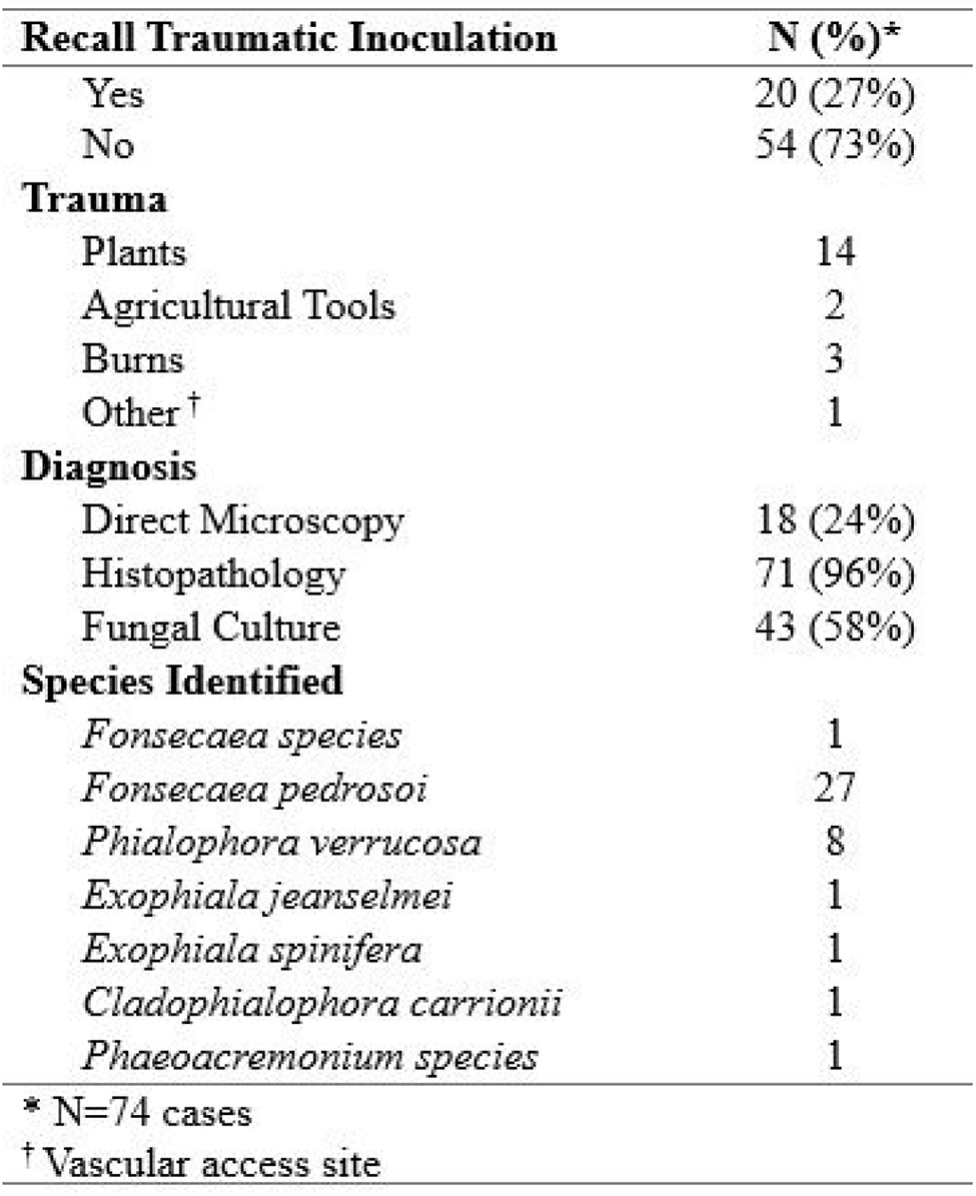
Chromoblastomycosis cases mode of inoculation and diagnostic outcome

**Methods:**

CBM cases were identified via literature search in Medline, Embase, EBSCO, and Scopus up to August 7, 2024. Search terms were “chromoblastomycosis” and “chromomycosis”. Inclusion criteria were muriform cells identified on direct microscopy or histopathology and acquisition or identification in the United States and Puerto Rico.

**Figure 1:**
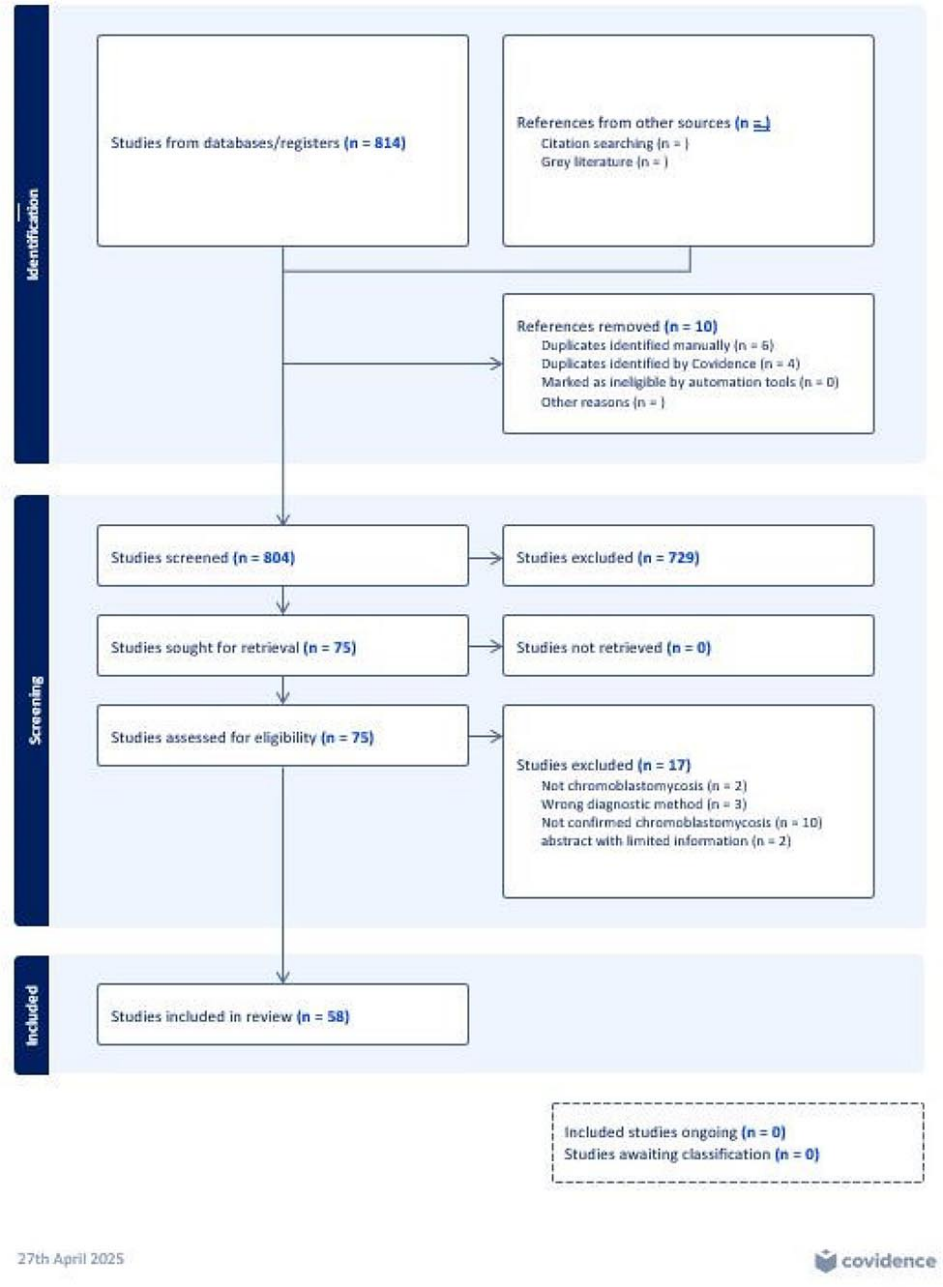
Preferred Reporting Items for Systematic reviews and Meta-Analyses (PRISMA) flowchart summarizing the study selection process, including identification, screening, eligibility, and inclusion of studies for analysis.

**Figure 2:**
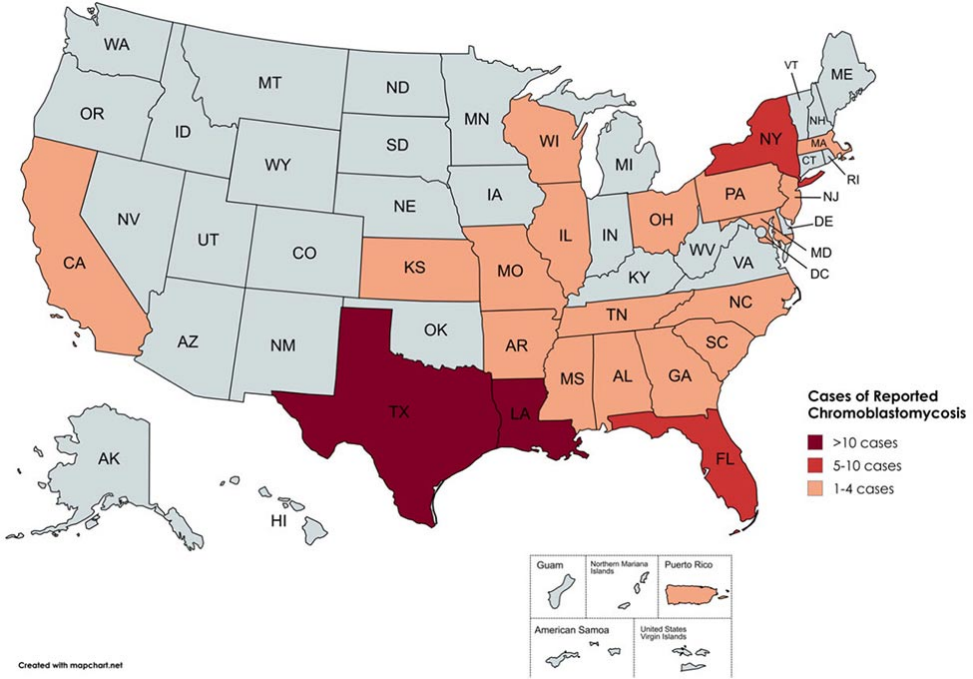
Map of the United States and Puerto Rico with prevalence of reported chromoblastomycosis cases by state.

**Results:**

We identified 58 articles (Figure 1) with 74 cases of CBM ever reported in the United States and Puerto Rico up to August 7, 2024 (Table 1). Mean age of diagnosis was 56.7 years; 62% were male. Nineteen percent had received systemic immunosuppressive therapy. Occupations included agricultural work (26%) and construction and manual labor (16%). Inoculation events were reported in 27% of cases including from plants (19%) and burns (4%). Cases were reported in 21 states, most commonly in Texas (19%), Louisiana (16%), and Florida (14%); over two-thirds of cases had no reported travel history (Figure 2).

Most cases were diagnosed via histopathology (96%) with fungal identification in 55% of cases, most commonly *Fonsecaea pedrosoi* (36%). Lesions presented on the upper (51%) and lower (38%) extremities as verrucous (47%) or nodular (39%); 11% had severe disease. Mean time from symptom onset to diagnosis was 5 years (3 weeks –22 years). Forty-one percent of patients received antifungal treatment, and 42% underwent surgical excision.

**Conclusion:**

CBM has a wide geographical distribution in the United States. Long delayed diagnoses highlight improvements needed in awareness and diagnostics. Prospective U.S. surveillance is critical to monitor for changes in CBM-causative fungi epidemiology and at-risk populations.

**Disclosures:**

Eva Rawlings Parker, MD, DTMH, L'Oreal Dermatologic Beauty: Advisor/Consultant|L'Oreal Dermatologic Beauty: Honoraria

